# Optimizing peripheral blood stem cells transplantation outcome through amend relapse and graft failure: a review of current literature

**DOI:** 10.1186/s40164-017-0082-5

**Published:** 2017-08-09

**Authors:** Saeed Mohammadi, Amir Hossein Norooznezhad, Ashraf Malek Mohammadi, Hajar Nasiri, Mohsen Nikbakht, Najmaldin Saki, Mohammad Vaezi, Kamran Alimoghaddam, Ardeshir Ghavamzadeh

**Affiliations:** 1 Hematology, Oncology and Stem Cell Transplantation Research Center, Tehran University of Medical Sciences, North Kargar Avenue, Tehran, 14117-13131 Iran; 2 Regenerative Medicine Research Center, Kermanshah University of Medical Sciences, Kermanshah, Iran; 3 Thalassemia and Hemoglobinopathy Research Center, Ahvaz Jundishapur University of Medical Sciences, Ahvaz, Iran

**Keywords:** Peripheral blood, Stem cells transplantation, Relapse, Graft failure

## Abstract

Allogeneic hematopoietic stem cell transplantation (allo-HSCT) has been considered as a valuable approach in treatment of numerous malignant and none malignant hematologic disorders. However, relapse and poor graft function (PGF) after allo-SCT remain to be controversial issues which may affect the transplantation outcome. Relevant articles were searched in MEDLINE database (2000–2016) using keywords and phrases: donor lymphocyte infusions, allogeneic stem cells transplantation, relapsed hematologic malignancies, booster schedules, cell dose, laboratory monitoring protocols and technical aspects of apheresis. Relapse of disease and PGF could be reduced via noting some main points such as choosing the suitable time and patient for donor lymphocyte infusion (DLI) and also determination of patients who ought to candidate for second allogeneic HSCT or for the use of stem cell boost. DLI and stem cell booster are promising treatment strategies noted in this review. Finally, this paper discusses indications and technical aspects of DLI and stem cell booster in hematological malignancies and emphasizes their therapeutic or pre-emptive potentials.

## Background

Over the past decades, allogeneic hematopoietic stem cell transplantation (allo-HSCT) was widely used as a curative choice for refractory and relapsed hematological malignancies in order to achieve a long-term survival [1, 2]. However, relapse and poor graft function (PGF) after transplantation still remained the major cause of treatment failure [3–5]. To enhance the effects of allo-HSCT, variable strategies have been established. According to this point, donor lymphocyte infusion (DLI) as a prophylactic and/or therapeutic strategy is an effective approach for patients with recurrent hematological malignancies after allo-HSCT through enhancement of graft versus leukemia (GVL) effect. GVL is known as a phenomenon helps patients to fight to their diagnosed disease mostly through T cells. In a study, inhibition of leukemia colonies growth was seen by donor T cells reactive to minor histocompatibility antigens [1]. Complications such as graft versus host disease (GVHD) and aplasia may happen after DLI. Prevalence of GVHD with the frequency of 50–60 after DLI, has no correlation with diagnosed disease. Although higher doses of T cells have more probability to occur. Interestingly, after GVHD a response to DLI as well as a disease free survival is predicted. Aplasia with less prevalence (20–40% after DLI) in comparison to GVHD has a mortality rate of 5% which its mechanism is still unclear. According to data the extent of residual host hematopoiesis seems to be the predictor of aplasia [1, 6, 7].

PGF, as another main cause of morbidity and mortality after allo-HSCT, is associated with infections, abnormal bleeding and blood transfusion related complications [8, 9]. Various therapeutic strategies could potentially improve PGF via stimulation of the already transplanted stem cells with growth factors [8, 9], re-transplant from same donor [10], or stem cell boost [11, 12]. In comparison to cited approaches, it seems that stem cell boost without chemotherapy or immunosuppressive conditioning could lead to improvement of survival outcomes [12–14]. Despite the wide use of DLI (therapeutic or pre-emptive) and booster as therapeutic strategies, it still seems to be difficult to achieve a consensus patient selection criteria, treatment schedules, cell dosage, and patient monitoring in different hematopoietic transplantation centers for relapsed and PGF transplanted patients. So, in this review we survey the recent publications in these fields with particular emphasis on mentioned problems and we also intend to prepare a practical guideline.

## Therapeutic donor lymphocyte infusion

This form of DLI has been indicated in patients with relapsed and progressed malignancies. Relapse is defined as the presence of bone marrow (BM) blast cells ≥5% or reappearance of blasts in peripheral blood and/or extramedullary regions [15, 16]. Major exclusion criteria are listed as uncontrolled infection, renal insufficiency (glomerular filtration rate <50 mL/min), hepatic impairment (bilirubin elevation to 4.5-fold above normal range), and malignant liver tumors [15, 17].

### Chemotherapy + DLI schedules

After chemotherapy with specific agent(s), in order to achieve complete remission (CR), DLI will be initiated based on the following strategies [18]. First, proper considered time of DLI suddested to be 1–2 week after chemotherapy in order to optimize the synergistic effect of chemotherapy and DLI [19]. Although the definite initial dosage is not confirmed, it has been established that high doses (>10 × 10^7^ CD3/kg) can induce more frequent GVHD. Also, previous efforts have not been successful in decreasing the risk of relapse occurrence and improvement of overall survival in the mentioned high doses. The initial dosage should be 1 × 10^6^ CD3/kg which is followed by logarithmic escalation [18–21]. In this strategy, the maximum and optimal cell dosage are different based on the type of malignancy. In chronic myeloid leukemia (CML) patients, the cell dosage less than 1 × 10^8^ T cells/kg is sub-optimal and doses above 4.5 × 10^8^ T cells/kg might lead to various complications [22–24]. In acute myeloid leukemia (AML) patients, any cell doses less than 1.5 × 10^8^ T cells/kg may lead to increased rate of failure. In patients with acute lymphoblastic leukemia (ALL), higher response rate was observed in studies using 1–2 × 10^8^ T cells/kg [23, 25, 26]. interestingly, the higher cell doses were associated with lower clinical response rates. In chronic lympho-proliferative disorders such as lymphomas, using DLI in 0.01–1.0 × 10^8^ T cells/kg dosage, higher response rates were observed [27–29]. Likewise, in multiple myeloma patients, it seems that there is not a well-defined relationship between cell dosages and response rates with DLI. Cell dosages ranged 0.01–8.2 × 10^8^ T cells/kg were used in different clinical trials [30–32].

### Azacitidine + DLI schedule

5-Azacytidine and DLI can induce long-term remissions in post allograft relapsed AML patients [33]. In this strategy, DLI was performed for transplanted AML and myelodysplastic syndromes (MDS) patients who were under chemotherapy after eight cycles of 5-azacitidine therapy (100 mg/m^2^/day, days 1–5, every 28 days). After every two cycles of 5-azacitidine, 1–5 × 10^6^ to 1–5 × 10^8^ CD3^+^cells/kg were infused [17, 34].

### Modified lymphodepletion (LD) + DLI schedule

This method is based on lymphocyte regulatory T cell (T reg) suppression during the LD process [35]. To minimize the risk of severe GVHD incidence, the following strategies could be considered. First, cyclophosphamide (CY) and fludarabine (FLU) should be administered at the doses of 600 mg/m^2^ on day 1 and 25 mg/m^2^/day on days 1–3, respectively. At the second step, donor lymphocytes were infused in a fixed high dose of 1 × 10^8^ CD3+ cells/kg, 48 h after the last FLU injection. Finally, patients who received chemotherapy along with the DLI (chemo-DLI) showed more frequent episodes acute GVHD, particularly in lower gastro-intestinal (GI) system [35–37].

### Technical tips for DLI procedure in chemotherapy DLI

**Fig. 1:**
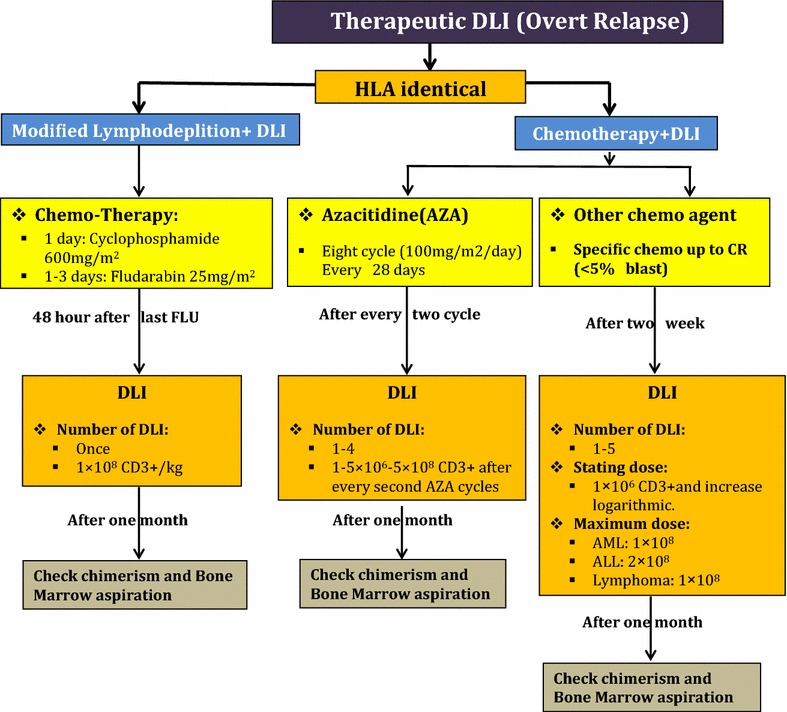
A summary of therapeutic DLI strategies

When low-dose of DL is requested (10^4^–10^5^ T-cells/kg), small aliquot of freshly collected blood from a related donor are needed. According to National Systems guidelines, if all tests for transfusion transmitted diseases were negative in sample collection time, this product could be readily used for patients. Usually, small aliquots of donor-recipients whole blood (1–2 mL), with major ABO incompatibilities do not seem to be life-threatening. When a high dose of DLI is requested, the product may be harvested through normal or large-volume leukapheresis [38, 39]. A summary of therapeutic DLI strategies is shown in Fig. 1.

## Pre-emptive/prophylactic donor lymphocyte infusion in HLA identical transplantation

Pre-emptive/prophylactic DLI is an acceptable choice especially for those patients who suffer from refractory or relapsed malignancies before transplantation, patients allo-grafted with active uncontrolled disease, patients who received transplantation in CR1 or CR1 >1, AML and ALL patients with high risk for relapse due to the positive minimal residual diseases [40] and also allo-graft transplanted patients with positive positron emotion tomography (PET) scan suffering from lymphoma. In these circumstances, DLI could be more effective because of the low tumor burden [20]. It seems that there is no advantage of administering DLI in the early stage of molecular relapse in patients who received allogeneic stem cell transplantation for CML [41–46]. Prophylactic DLI has not been administered in several situations including: the onset of GVHD, uncontrolled infections, early relapse, engraftment failure and organ failure [47]. In this strategy, immunomodulation consists of early reduction of immunosuppression medication which was then followed by DLI. In patients with appearance of GVHD, tentative tapering of immunosuppressive therapy should be considered. On the other hand, in patients with no incidence of GVHD, immunosuppression should be discontinued and donors would be eligible for lympho-apheresis [41–46].

### Treatment schedule for preemptive/prophylactic un-modified DLI based on molecular markers in HLA identical transplantation

In this strategy, Wilms tumor-1 (WT1) gene expression exceeding 100 copies/10^4^ abelson murine leukemia-1 (ABL1) in bone marrow (BM) or WT-1 ≥5 copies/10^4^ ABL1 in peripheral blood, with BM in remission status (<5% blasts simultaneously with morphology and cytometry symptoms) is surrogate for starting DLI [45, 48]. In absence of GVHD for HLA identical siblings transplantation, the starting dosage is 1 × 10^6^/kg CD3+ cells and subsequently, half log increments every 30–60 days until MRD becomes negative. In unrelated transplant recipients, the initial dosage is 1 × 10^5^/kg CD3+ cells, along with half log increments every 30–60 days until MRD becomes negative. If GVHD developed after DLI, the program should be ceased until it would be completely suppressed [44–46].

### Treatment schedule for preemptive/prophylactic un-modified DLI for high risk patients

This DLI strategy has been applied in two clinical trials for ALL patients. The median time and CD3+ cell dosage after HSCT are 60–185 days and 1.5–3 × 10^6^ cells, respectively [49, 50].

### Modified prophylactic DLI (mpDLI) or G-CSF primed in HLA identical transplantation

This strategy might take advantage by using donor’s G-CSF-mobilized peripheral blood progenitor cells (GPBPCs) instead of their steady lymphocytes. In this method, infused cells have super anti-leukemia effects. Likewise, this approach may be accompanied by a reduction in infusion-related GVHD and also it is rarely challenged by the risk of pancytopenia [51, 52]. MRD-based modified DLI intervention is associated with transplant outcomes improvement. MRD could be evaluated using qualitative nested PCR for immunoglobulin heavy chain (IgH) VDJ, TCR gene rearrangement, WT1 expression and leukemia associated immunophenotypes (LAIP). Combination of these biomarkers is more sensitive in relapse prediction than each of WT1 or LAIP separately after transplantation [48, 51, 52]. This form of DLI is indicated after HLA-identical HSCT for patients with unfavorable cytogenetic abnormalities and also acute leukemia in more than CR1 status or in the non-remission state. Likewise, this strategy is indicated in CML in both accelerated phase (AP) and blast phase (BP) as well as myeloproliferative disorders with unfavorable cytogenetic abnormalities such as 8p11 [50–54] (Table 1).

**Table 1:** Summary of trials mentioned in current review

Type of DLI	Authors	Case number	Mean age of patients (years)	Pre-DLI treatment(s)	Diagnosis	DLI modality	T cell dosage	Series of DLI (mean)	GVHD	Response rate (CR)
Therapeutic DLI										
Lymphodepletion (LDP)	Warlick et al. [7]	35	51	LPD	ALL, AML, MDS, NHL, CLL, MM	Unstimulated leukapheresis	0.5 × 10^8^/kg 1 × 10^8^/kg	1	II–IV (25%) II–IV (66%)	CR 45% CR 53%
	Guillaume et al. [36]	18	45	Escalation LDP	ALL, AML, MDS, NHL, HD, MM	Not mentioned	1–5 × 10^7^/kg	1–3	II–IV (29.4%)	CR 38.9%
With pharmacotherapy	Schroeder et al. [16]	154	55	5-Azacytidine	AML, MDS	Not mentioned	31.2 × 10^6^/kg (mean)	1–3	Acute (23%)	CR 27%
	Schroeder et al. [17]	30	55	Azacitidine	AML, MDS	Dose-escalating donor lymphocyte	1.5 × 10^6^/kg–1.5 × 10^8^/kg	1–4	II–IV (30%)	CR 38.1%
G-CSF-primed	Yan et al. [48]	50	22	Chemotherapy	ALL, AML	G-CSF primed	0.9–4.2 × 10^8^/kg	1	II–IV (62.7%)	CR 32%
Prophylactic	Wang et al. [47]	123	40	No treatment	ALL, AML	Prophylactic (G-CSF primed type)	1.8 × 10^8^/kg	Not mentioned	II–IV (17%)	Not mentioned
	Kumar et al. [39]	18	60	Immunosuppressive therapy	ALL, AML, MDS	Prophylactic ex vivo costimulated T cells	1–10 × 10^7^/kg	1–2	Acute (27.8%)	CR 4 of 18

*AML* acute myeloid leukemia, *ALL* acute lymphoblastic leukemia, *CML* chronic myeloid leukemia, *CLL* chronic lymphoblastic leukemia, *DLI* donor lymphocyte infusion, *G-CSF* granulocyte-colony stimulating factor, *HD* Hodgkin disease, *MDS* myelodysplastic syndrome, *MM* multiple myeloma, *MPN* myeloproliferative syndrome, *NHL* non-Hodgkin lymphoma

### Treatment schedule for (mpDLI) or G-CSF primed prophylactic DLI (without MRD monitoring)

Short-term immunosuppressive agents (cyclosporine A or methotrexate (MTX) 10 mg/week for 2–4 weeks) are in use for prevention of DLI associated GVHD. DLI could be considered for use from days 45 to 120 after transplantation in patients without recurrence of leukemia and GVHD. In some conditions such as onset of GVHD, uncontrolled persistent infections longer than 60 days and postponement of consent, it has been suggested to avoid performing prophylactic DLI. 1 × 10^8^ MNCs/kg seems to be a proper initial cell dosage for DLI in patients who have received DLI before day 90 after transplantation. Cyclosporine A (CsA) should be continued for 2 weeks and it tapered within 4 weeks and discontinued after then if no DLI-associated GVHD occurs. In patients who received DLI after day 90 of transplantation, all immunosuppressive agents should be stopped for at least 2 weeks before DLI infusion in in order to prevent GVHD. These patients should take oral CsA or MTX 10 mg/week (single dose) for 2–4 weeks after DLI intended for prevention of DLI-associated GVHD [47, 53].

### Treatment schedule for (mpDLI) or G-CSF primed prophylactic DLI (MRD base)

In this strategy, WT1 gene expression more than 100 copies/10^4^ ABL1 in BM or WT1 ≥5 copies/10^4^ ABL1 in peripheral blood, together with bone marrow remission (<5% blasts), and concordance of morphology and flow cytometry (FCM) is surrogate for starting DLI. Positive FCM is defined as >0.001% of cells with a leukemia-associated immunophenotypes (LAIP phenotype in ≥1 BM samples after transplantation) [48]. The immune intervention before DLI is determined as a post transplantation immune suppression which should be immediately tapered and then discontinued in patients with MRD+ ≤100 days after transplantation. Immune suppression was immediately discontinued in MRD+ patients in whom more than 100 days have been passed from their transplantation. In HLA identical siblings recipients, DLI should be started with 1 × 10^8^/kg MNC and consequently, with half log increments every 30–60 days until MRD becomes negative, in the absence of GVHD. In unrelated transplant recipients, DLI should be started with 1 × 10^8^/kg MNC and subsequently with half log increments every 30–60 days until MRD becomes negative. If GVHD develops after DLI, the program should be stopped until it resolves [48, 55]. After DLI, patients should receive immunosuppressive medications such as CsA or MTX in order to prevent GVHD. CsA is usually started at the dosage of 2.5 mg/kg/day and it must be adjusted to maintain plasma concentration of >100 ng/mL. MTX should be started at 10 mg IV on days 1, 4, 8, and continued weekly for 2–6 weeks. In patients receiving DLI from an HLA-identical related donor, GVHD prophylaxis should be received for 2–4 weeks. In patients receiving DLI from an HLA-matched unrelated or HLA-haploidentical donor, GVHD prophylaxis regimen should be prescribed for 4–6 weeks at the discretion of the well trained physicians which usually depends on patient’s GVHD status after DLI [48, 55].

### Treatment schedule for pre-emptive/prophylactic unmodified DLI for mix chimerism (MC) patients

This DLI strategy has been applied in three clinical trials for lymphoma, AML and MDS patients. The median time after HSCT ranged from 35 to 287 days and the median CD3+ cell dosage ranged from 1 × 10^7^ to 5.7 × 10^7^ in these studies [42, 56, 57].

### Technical tips for DLI procurement

G-CSF stimulated peripheral blood stem cells should be harvested through leukopheresis and DLI might be obtained from negative fraction of CD34+ hematopoietic stem cells selection. Based on many published reports, differences among last generation devices are minimum; as a result, most of cell separator devices (such as Spectraoptia^®^ and Fresenius Com. Tec^®^) are able to collect highly enriched lymphocyte fraction with very low erythrocyte pollution and in some cases reduced platelet content. These aliquots are therefore maintained frozen until thawing to infusion [58, 59].

**Fig. 2:**
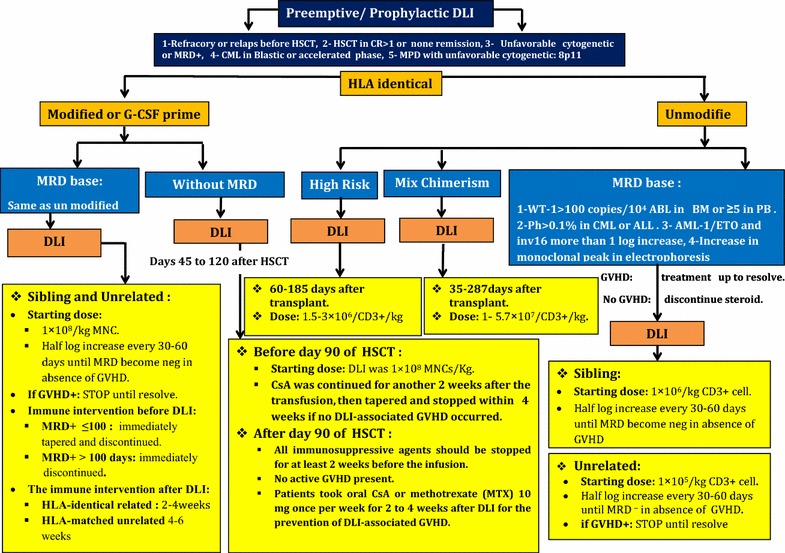
A summary of prophylactic/pre-emptive DLI strategies in HLA identical transplantation

A summary of prophylactic/pre-emptive DLI strategies in HLA identical transplantation is shown in Fig. 2.

## DLI for haploidentical transplantation

### Therapeutic donor lymphocyte infusion (t-DLI) in haploidentical transplantation

#### Chemothrerapy-DLI with Baltimore platform with post-transplant cyclophosphamide (PT-Cy)

PT-Cy has been used for relapsed patients after haploidentical HSCT. The initial used CD3+ dosage is 1 × 10^5^/kg of recipient and all immunosuppression agents should be discontinued [60, 61].

### Pre-emptive/prophylactic DLI (pDLI) in haploidentical transplantation

#### MRD based un-manipulated DLI (without GCS-F)

This strategy is applied for AML patients with MRD relapse. Firstly, patients receive a low dosage of CD3+ (1 × 10^3^/kg) and then a gradually increased dosage up to 1 × 10^5^/kg–1 × 10^6^/kg would be used [61, 62].

#### Treatment schedule for G-CSF primed prophylactic DLI (without MRD monitoring)

Short-term immunosuppressive agents [CsA or MTX 10 mg/week (single dose) for 2–4 weeks] should be administered in order to prevent DLI associated GVHD. DLI could be considered from days 45 to 120 after transplantation in patients without recurrence of leukemia and GVHD [53]. The principal reasons for this delayed prophylactic DLI administration are introduced as GVHD occurrence, uncontrolled infections (lasting longer than 60 days), and postponement of consent. As a key point, clinicians should remember to eradicate serious infections before DLI accomplishment. Furthermore, no serious organ failure should be detected. The initial cell dosage for DLI is 1 × 10^8^ MNC/kg for patients who are candidate to receive DLI before day 90 after transplantation. Also, CsA should be continued for 2 additional weeks. After that, CsA should taper and finally be discontinued within 4 weeks if no DLI associated GVHD occures. For patients who have received DLI after day 90, all immunosuppressive agents should be stopped for at least 2 weeks before the DLI infusion when no active GVHD is present. Oral CsA or MTX 10 mg/week (single dose) for 2–4 weeks after DLI is advised in order to prevent DLI-associated GVHD in these patients [47, 51–54, 60, 61].

#### Technical tips for DLI procurement

**Fig. 3:**
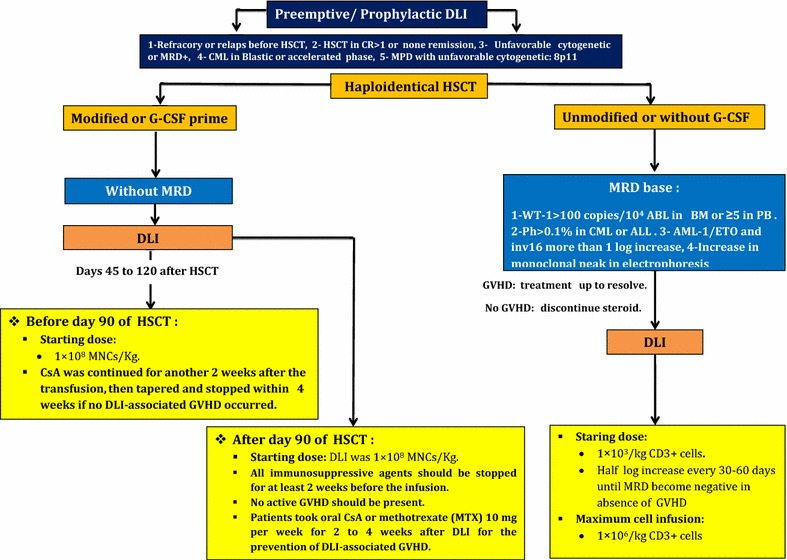
A summary of prophylactic/pre-emptive DLI strategies in HLA haploidentical transplantation

G-CSF stimulated peripheral blood stem cells should be harvested through leukopheresis and DLI might be obtained from the negative fraction of the CD34+ hematopoietic stem cell selection. These aliquots are preserved and thawed at different intervals for infusion [58, 59]. A summary of prophylactic/pre-emptive DLI strategies in HLA haploidentical transplantation is shown in Fig. 3.

## Evaluation of response to DLI

The best response rates are observed, respectively, in patients with CML, lymphomas, multiple myeloma and acute leukemia. The responses in patients suffering from CML are durable in comparison to other patients with other malignancies in chronic phase. CML patients show better response rather in accelerated/blastic phase. Patients with only molecular and cytogenetic relapses always went into remission with DLI, while in patients with chronic phase, hematologic relapse remission rate is about 75%. Continuing DLI for patients with merely cytogenetic/molecular relapse may be helpful to achieve the least remission rates. In patients with lymphoma, highest response rates have been observed in indolent lymphomas, while aggressive lymphomas contained to have lower response rates. Significant responses can be attained in more aggressive lymphomas; however, chemotherapy seems to be prerequisite for success [1, 63–65].

## Cell booster in poor graft function transplantation

### Booster indications

Booster has indication in the following situations:

1. Primary graft failure (PGF) is defined as cytopenia occurrence in at least two hematopoietic lines beyond the 21st day after transplantation. In this circumstance, neutrophil count ≤1.5 × 10^9^/L, platelet count ≤30 × 10^9^/L and Hb ≤8.5 g/dL. Engraftment of neutrophils is determined as the first of three consecutive days when absolute neutrophil count (ANC) was >0.5 × 10^9^/L without G-CSF (5 µkg/kg body weight) stimulation. Engraftment of platelets is defined in the first of three consecutive days when the platelet count is ≥20 × 10^9^/L (independent from platelet substitution) [12–14, 66, 67].
2. Secondary graft failure is defined as BM hypoplasia (<10% cellularity) after engraftment which in this situation patient requires frequent (more than once a week) platelet transfusions. Among other criterias for secondary graft failure are ANC of less than 0.5 × 10^9^/L without growth factor therapy beyond day 60 in presence of full donor chimerism and in absence of severe GVHD, CMV reactivation, relapse or drug-related myelo-suppression [12–14, 66, 67].

### Treatment schedule for booster

**Fig. 4:**
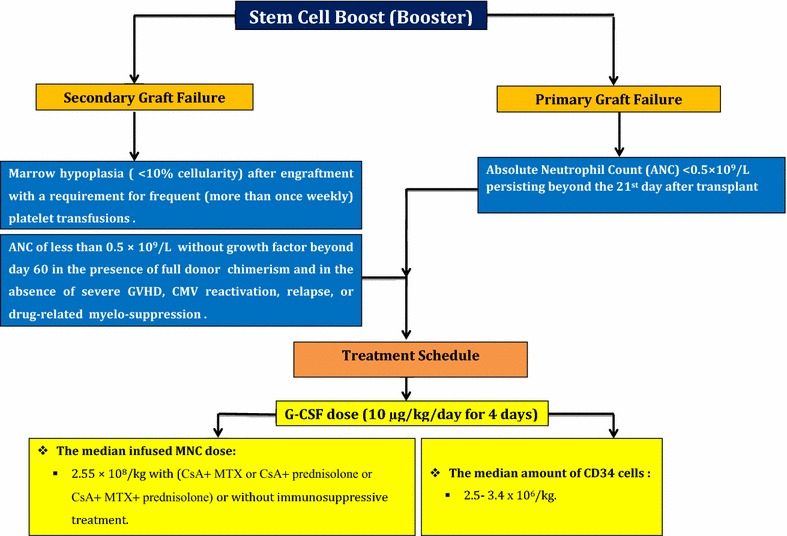
A summary of stem cell boost (booster) strategy for poor graft function after allogeneic transplantation

PBSC donors should be stimulated with G-CSF (10 µg/kg/day for 4 days) prior to leukapheresis. Based on recent data, the median amount of CD34‏ cells is suggested to be 3.4 × 10^6^/kg for a successful leukopheresis. Also, the median infused MNCs dosage is 2.55 × 10^8^/kg with (CsA + MTX or CsA + prednisolone or CsA + MTX + prednisolone) or without immunosuppressive treatment [4, 12, 13, 66, 67]. A summary of stem cell boost (booster) strategy for poor graft function after allogeneic transplantation is shown in Fig. 4.

## Conclusions

DLI and booster are two main therapeutic opportunities for precluding or treating relapse and PGF of hematologic malignancies after allo-HSCT. Likewise, DLI has been used in advanced-stages of malignancies and augmenting immune reconstitution, although, GVHD considered as a major complication of using DLI in clinic. Hence, a good strategy would be to enhance GVL effect and in the intervening time to minimize GVHD. On the most important way to differentiate between GVHD and GVT is DLI using tumor specific donor lymphocytes [19]. Also, it has been showed that the dose-escalating strategy could use as a method for management of balance between GVL and GVHD after DLI [17].

The recent presented data on DLI and booster studies certainly provide valuable information for optimizing these therapeutic strategies in order to be applied in different clinical settings. However, the protocols for prophylactic and therapeutic DLIs may require some modifications. Despite the recent published data on DLI and booster up to now, it seems the dosage of infused cells, the timing and frequency of administration, and the continuity of treatment still need to be optimized for any given clinical setting.
